# Attitudes among healthcare professionals towards ICT and home follow-up in chronic heart failure care

**DOI:** 10.1186/1472-6947-12-138

**Published:** 2012-11-28

**Authors:** Anna Gund, Kaj Lindecrantz, Maria Schaufelberger, Harshida Patel, Bengt Arne Sjöqvist

**Affiliations:** 1 Department of Signals and Systems, Chalmers University of Technology, Gothenburg, 412 96, Sweden; 2 School of Technology and Health, KTH - Royal Institute of Technology, Stockholm, 100 44, Sweden; 3 Department of Clinincal Science, Intervention and Technology, Karolinska Institutet, Stockholm, 141 86, Sweden; 4 Department of Emergency and Cardiovascular Medicine, Institute of Medicine, Sahlgrenska Academy at University of Gothenburg, Gothenburg, 416 85, Sweden; 5 Institute of Health and Care Sciences, Sahlgrenska Academy at University of Gothenburg, Gothenburg, 405 30, Sweden

## Abstract

**Background:**

eHealth applications for out-of-hospital monitoring and treatment follow-up have been advocated for many years as a promising tool to improve treatment compliance, promote individualized care and obtain a person-centred care. Despite these benefits and a large number of promising projects, a major breakthrough in everyday care is generally still lacking. Inappropriate organization for eHealth technology, reluctance from users in the introduction of new working methods, and resistance to information and communication technology (ICT) in general could be reasons for this. Another reason may be attitudes towards the potential in out-of-hospital eHealth applications. It is therefore of interest to study the general opinions among healthcare professionals to ICT in healthcare, as well as the attitudes towards using ICT as a tool for patient monitoring and follow-up at home. One specific area of interest is in-home follow-up of elderly patients with chronic heart failure (CHF). The aim of this paper is to investigate the attitudes towards ICT, as well as distance monitoring and follow-up, among healthcare professionals working with this patient group.

**Method:**

This paper covers an attitude survey study based on responses from 139 healthcare professionals working with CHF care in Swedish hospital departments, i.e. cardiology and medicine departments. Comparisons between physicians and nurses, and in some cases between genders, on attitudes towards ICT tools and follow-up at home were performed.

**Results:**

Out of the 425 forms sent out, 139 were collected, and 17 out of 21 counties and regions were covered in the replies. Among the respondents, 66% were nurses, 30% physicians and 4% others. As for gender, 90% of nurses were female and 60% of physicians were male. Internet was used daily by 67% of the respondents. Attitudes towards healthcare ICT were found positive as 74% were positive concerning healthcare ICT today, 96% were positive regarding the future of healthcare ICT, and 54% had high confidence in healthcare ICT. Possibilities for distance monitoring/follow-up are good according to 63% of the respondents, 78% thought that this leads to increased patient involvement, and 80% thought it would improve possibilities to deliver better care. Finally, 72% of the respondents said CHF patients would benefit from home monitoring/follow-up to some extent, and 19% to a large extent. However, the best method of follow-up was considered to be home visits by nurse, or phone contact.

**Conclusion:**

The results indicate that a majority of the healthcare professionals in this study are positive to both current and future use of ICT tools in healthcare and home follow-up. Consequently other factors have to play an important role in the slow penetration of out-of-hospital eHealth applications in daily healthcare practice.

## Background

The field of eHealth [1,2], as well as the use of information and communication technology (ICT) within healthcare, is constantly growing. Today, many tasks previously performed with pen and paper are performed using computers and networks. For example, electronic health records (EHR) and electronic prescribing (ePrescribing) are commonly used. In Sweden, as an example, more than 80% of all prescriptions are in electronic form [3], and 100% of primary care units have access to EHR [4].

eHealth applications for out-of-hospital monitoring and treatment follow-up have been advocated for many years as promising tools to improve treatment compliance, promote individualized care and obtain a person-centred care. As a result this is expected to lead to improved treatment outcome, patient safety and a more efficient use of available resources.

Despite these benefits of out-of-hospital monitoring and treatment follow-up, and the large number of promising projects, a more general breakthrough in everyday care and practice is still lacking. This could be explained by several factors such as the lack of an appropriate organization for the new technology, reluctance from the users to introduce new ways of working, and negative opinions regarding ICT and the proposed methods in general. When introducing new ICT based eHealth applications there are attitude barriers causing delays, or interruptions, in implementation [5-9]. To be able to continue the development, and increase the adoption rate, of ICT in healthcare it is therefore of importance to study the general opinions among healthcare professionals with regard to ICT support in out-of-hospital care.

In this study we have chosen to target an important patient group often considered for out-of-hospital eHealth applications, namely patients suffering from chronic heart failure (CHF) [10]. This is a disorder which affects approximately 2% of the population as a whole in the western world [11,12], and as many as 10–20% in the population aged 70 or more [13]. Moreover, these numbers are expected to grow as a consequence of demographic changes and improved healthcare. This group is also associated with significant healthcare costs. If eHealth solutions can help individuals to stay healthier and keep better control of their disease it would be a large benefit for both patient and society.

There are several current projects working with eHealth solutions for CHF patients, in terms of structured telephone support as well as more technologically advanced telemonitoring systems. These show very different results in terms of both healthcare outcome as well as user satisfaction, which e.g. is demonstrated in a review by Inglis et al. [14]. Chaudhry et al. found no significant difference between a group assigned telemonitoring and a control group [15], while Dendale et al. show the opposite results [16]. Patient acceptance to this type of care was shown to be positive by e.g. Venter et. al and Seto et al. [17,18]. The project Care@Distance, of which this study is a part [19], aims at using a generic Internet-based system for disease management of patients in out-of-hospital care. Preliminary results indicate a positive reaction from users, but also some difficulties related to practical clinical introduction.

We have limited the study to Sweden only, but we believe that results from Sweden translate well to most countries in the western world. As Internet and computer usage is among the larger in otherwise comparable countries [20-22], a study in Sweden may also serve as a predictor for the situation in these other countries.

The aim of this study was to investigate the general attitudes towards, and confidence in, ICT in healthcare today and in the future among healthcare professionals working with CHF patients. Another aim is to study the attitudes towards home follow-up/distance monitoring of these patients. Also, it is of interest to see whether CHF patients are considered suitable for home follow-up by the healthcare professionals, and which methods for patient follow-up that are considered most appropriate. Moreover, we wanted to see whether any differences could be found between various groups of respondents.

Similar studies on attitudes towards healthcare ICT have been collected by Ward et al. in a review from 2008 [7]. However, surprisingly considering the large amount of eHealth systems for CHF patients, none of these studies are focused on healthcare professionals working with CHF care. Instead they cover healthcare professionals’ attitudes towards ICT in healthcare in general, or in areas such as other specialities (e.g. paediatrics, pharmacy or education in medicine), certain geographical areas, or regarding specific ICT tools (e.g. introduction of a new EHR). Moreover, since knowledge in ICT is rapidly growing among the general population, the views on healthcare ICT could differ substantially depending on when and in which country the study is performed. Therefore, the results in this study could contribute to the already existing knowledge base, giving a better understanding to the difficulties in implementing eHealth solutions and healthcare ICT into clinical practice.

## Method

In total, 425 questionnaires were sent out to 85 hospital departments responsible for CHF care, e.g. cardiology or medicine departments, in all 21 counties and regions in Sweden. The number of departments in each county or region depends on their size. The largest regions, with almost 35% of the departments, are Västra Götaland, Skåne and Stockholm. One department, Sahlgrenska University Hospital/Östra, in Västra Götaland region was excluded because of risk for project collaboration bias.

Envelopes containing 5 printed questionnaires with an information letter and a self-addressed (stamped) envelope were addressed to the head of department at each of the 85 departments along with a letter requesting their help to administer the forms to relevant personnel. A link was included in each questionnaire for those who preferred to answer the form on Internet. After 4 weeks a reminder was sent out to each department by post.

This method of distribution was chosen since it was impossible to get access to an address list or other source containing relevant information on staff working with care of CHF at hospitals in Sweden. Obviously, this is not an optimal method of distribution. Bias, such as the head of department choosing respondents that have a predisposed attitude towards ICT, is one big issue. Also, as the amount of employees at each department is unknown, there is no way to be sure that 5 questionnaires is an appropriate amount. However, in order to reach a large population, both in terms of amount of respondents and geographical spread, this method was considered adequate for the purpose of the study.

Anonymity of the respondents was assured by not including name or other personal information in the questionnaire. Moreover, hospital affiliation was not included to further ensure anonymity. The respondents were urged to answer all questions, but they were not required to.

The questionnaire, which was written in Swedish, included 33 questions divided into 4 categories: background, attitudes to ICT tools in healthcare, opinions on follow-up at home, and other. In the category “background”, questions were asked on age, sex, occupational title, county, and computer experience at work and at home. The category “attitudes to ICT-tools in healthcare” asked general questions regarding ICT as a tool in healthcare today and in the future, possibilities of patient monitoring at a distance, whether distance monitoring can result in better self-care, provide healthcare professionals with possibilities to administer better care, reduce costs and save time, and general reliance to ICT as a tool in healthcare.

Further, in “opinions on follow-up at home” questions were asked on if patients with CHF were appropriate for follow-up at home, which patients that were best suited, and which were the best ways of performing the follow-up. Free text fields made it possible for the respondents to give additional information

In the last category, “Other”, the respondents were asked if they had any prior knowledge in the area or had any additional information they wanted to share. The respondents were also given the possibility to leave contact information in case they would like further information, or were interested in participating in future trials. This last question was separated from the others and put in a separate pile before analysis in order to preserve anonymity.

Approximately 10 weeks after sending out the questionnaires, the retrieved results were compiled and analysed. Responses received after this time were archived and not included in the study. Due to the uncertainty in the method of dispatch, no conclusions will be drawn on the general opinions of the population as a whole. Moreover, since the intention of this study was not to perform a hypothesis test advanced statistical analysis methods were not used. Instead, we present data in diagrams and tables, with percentages of the total. Due to rounding errors, some results will add up to more than 100%. All questionnaires can be retrieved from the project homepage [23].

In order to analyse the data, the occupation was divided into groups: physicians and nurses, men and women. The group “physicians” consisted of cardiologists, other specialist physicians and GP (General Practitioner). Nurses specialized in heart diseases as well as in other specialties, registered nurses and assistant nurses made up the “nurse” group. Those who had entered both “head of department” and “physician” were allocated to the physician group. The other duplicates did not affect the physician and nurse groups since they had chosen both “cardiology/heart” and “other”, but stayed within the group of physician/nurse.

## Results

### Background information

Among the 425 forms sent out 139 replies (33%) were received from 17 of the 21 counties and regions in Sweden. The share of collected questionnaires in relation to dispatched questionnaires varied greatly among the regions from 16% to 80%. Out of the answers 133 were returned by post and 6 were answered through the Internet form.

The background information of the respondents can be seen in Table 1. A majority of the respondents were female. This was expected as more than 80% of people working in healthcare in Sweden are female [24], and the form was sent out to all categories of healthcare workers.

**Table 1:** Background information of respondents

**Characteristics**	**N**	**%**
**Gender**		
Male	34	24%
Female	101	73%
Not specified (NS)	4	3%
**Occupation**		
Head of department	5	4%
Specialized physician, cardiology	31	22%
Spec. physician, other	10	7%
GP	1	1%
Spec. nurse, heart	56	40%
Spec. nurse, other	3	2%
Nurse	23	17%
Assistant nurse	10	7%
Other	5	4%

The results on primary occupation adds up to more than 100% as 5 respondents noted two titles (e.g. both head of department and physician). When grouping the results into the two groups “Nurses” and “Physicians” the numbers were adjusted as described in the Methods section. After this adjustment the results show that most respondents, 66% (N=91), were nurses. More specifically, the largest group was nurses specialized in heart diseases. Physicians accounted for 30% (N=40) of the answers.

Table 2 shows the gender distributions among physicians and nurses. As can be seen a majority of the physicians were male, and a large majority of the nurses were female. These results match numbers from Swedish national statistics, which show a large gender difference between physicians and nurses in Sweden [25].

**Table 2:** Gender distribution among physicians and nurses

	**Male (N=34)**		**Female (N=101)**		**Not specified (N=4)**	
	**N**	**%**	**N**	**%**	**N**	**%**
**Physicians (N=40)**	24	60%	14	35%	2	5%
**Nurses (N=91)**	7	8%	82	90%	2	2%

As expected, considering the amount of healthcare ICT used in Sweden [3,4], an overwhelming majority of the respondents used computers several times a day, as can be seen in Table 3. In order to investigate the general ICT interest among the respondents, we instead decided to study the use of computers at home, shown in Table 4. Computer usage at home could indicate an ICT interest as they choose to use computers outside their work. At home the use of computers varied more than usage at work, but still two thirds of the respondents answered that they use computers in their homes at a daily basis. The occupational difference was larger for computer difference at home compared to computer usage at work.

**Table 3:** Computer usage at work among the respondents

	**Never**		**Weekly**		**Daily**		**Several Times per Day**		**Largest part of Day**	
	**N**	**%**	**N**	**%**	**N**	**%**	**N**	**%**	**N**	**%**
**Total (N=139)**	1	1%	1	1%	1	1%	37	27%	99	71%
**Physicians (N=40)**	0	0%	0	0%	0	0%	10	25%	30	75%
**Nurses (N=91)**	1	1%	1	1%	1	1%	25	27%	63	69%

**Table 4:** Computer usage at home among the respondents

	**Never**		**Rarely**		**Weekly**		**Daily**		**Several Times per Day**	
	**N**	**%**	**N**	**%**	**N**	**%**	**N**	**%**	**N**	**%**
**Total (N=139)**	1	1%	14	10%	31	22%	56	40%	37	27%
**Physicians (N=40)**	0	0%	1	3%	4	10%	15	38%	20	50%
**Nurses (N=91)**	1	1%	13	14%	25	27%	39	43%	13	14%

### Attitudes to ICT tools in healthcare

**Figure 1:**
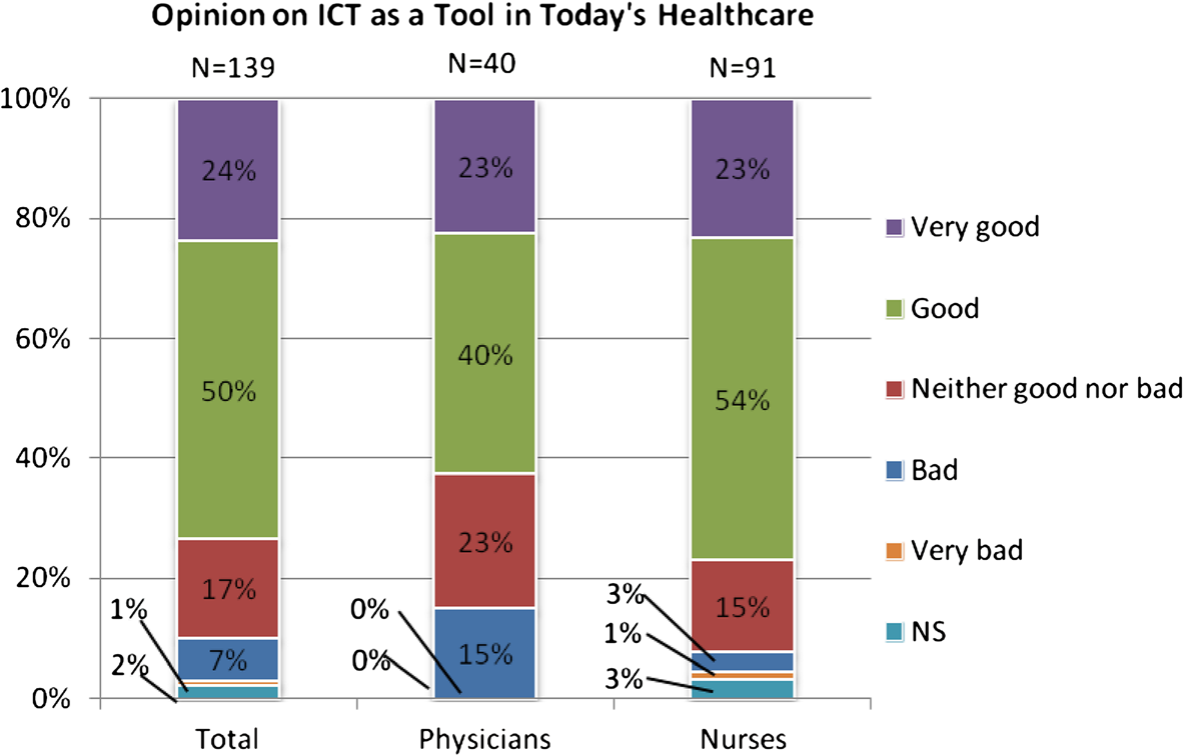
Attitudes among respondents towards healthcare ICT today.

**Figure 2:**
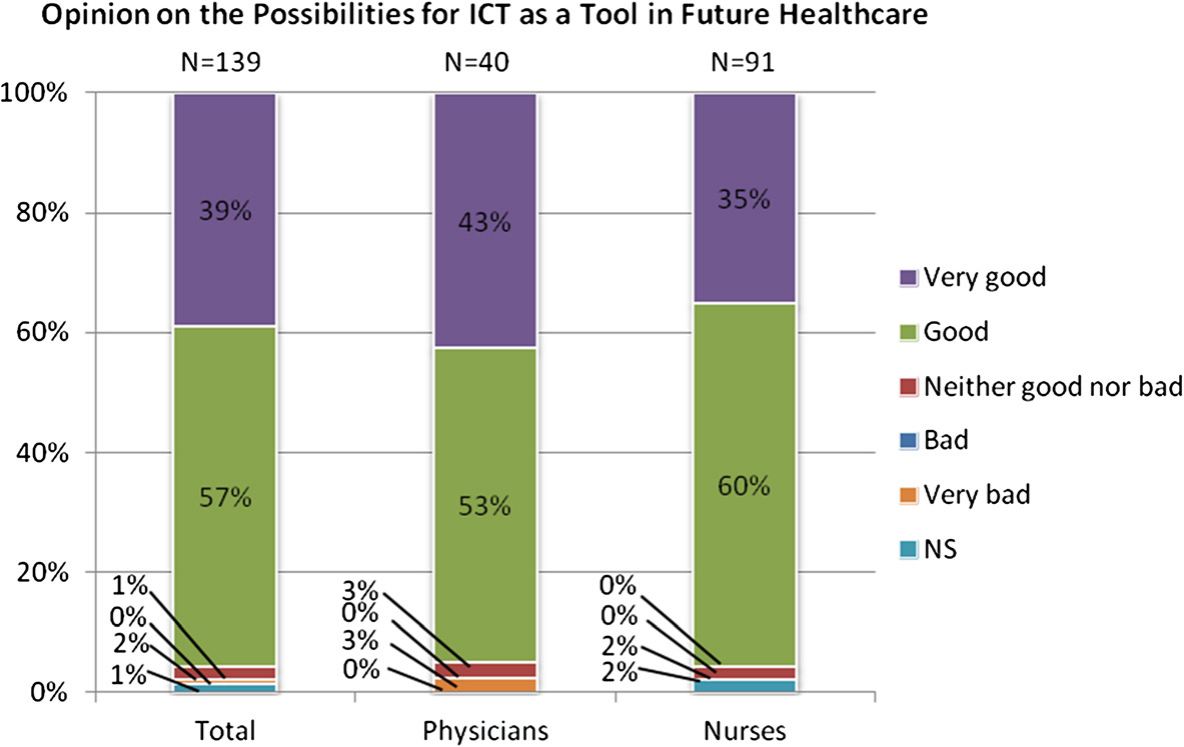
Attitudes among respondents towards ICT as a tool in healthcare in the future.

**Figure 3:**
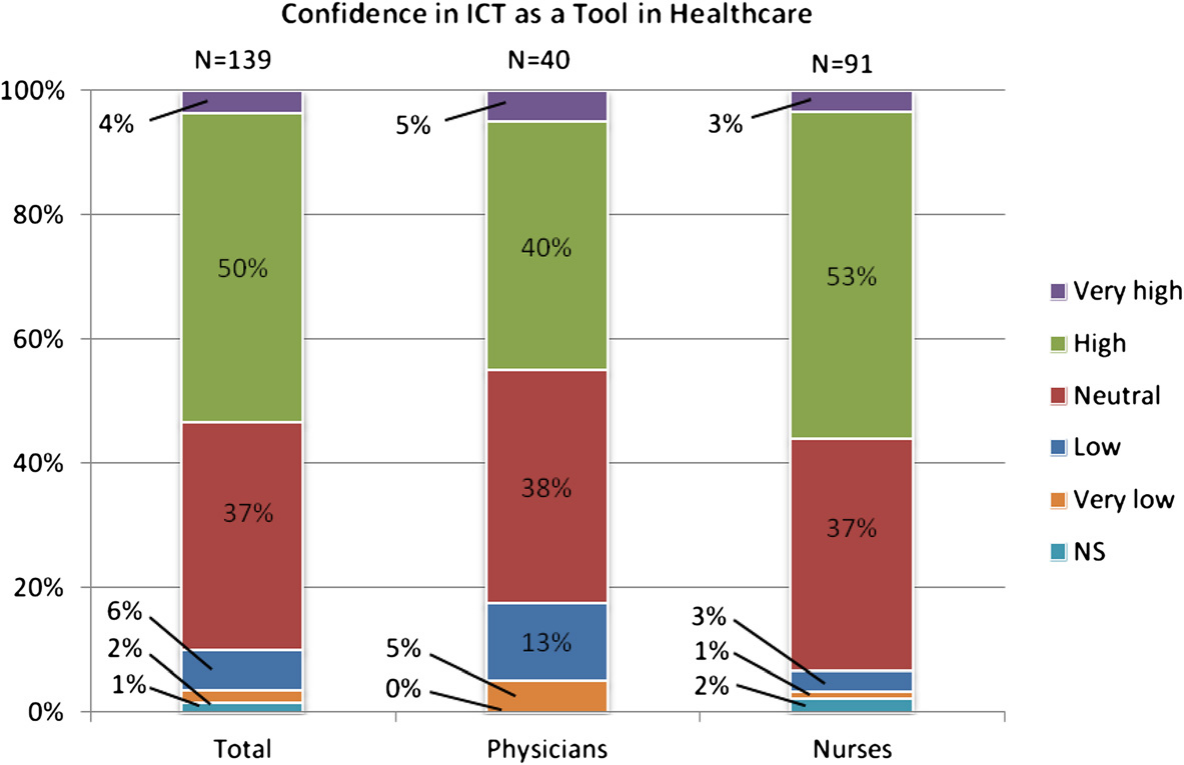
Confidence in ICT as a tool in healthcare among respondents.

Figures 1 and 2 show the attitudes towards healthcare ICT today and in the future. In both cases a majority of the responders had positive opinions, and very few had negative opinions. Moreover, the outlook on future possibilities was more positive than the view on today’s healthcare ICT. Confidence in ICT as a tool in healthcare, as shown in Figure 3, was more neutral as a bit more than a half of the respondents had high confidence. Again, very few had low confidence in healthcare ICT, but a larger part was neutral compared to the previous two questions.

Besides attitudes towards and confidence in ICT in healthcare, questions regarding cost and time were also covered in this section. More than half, 57%, thought ICT tools lower costs, while only 6% thought ICT tools increase costs in healthcare. Costs are unaffected according to 29% of the respondents. As for time aspects, 64% of the respondents thought that ICT tools in the future will save time, while only 4% thought it will take more time. ICT tools will not affect time aspects according to 26%.

### Attitudes towards distance monitoring and home follow-up of patients

Before asking about the attitudes toward distance monitoring and home follow-up of patients, it could be of interest to know how many who have experience in the matter. Out of the respondents, 14% claim they had experience and 85% had no experience (1% NS) of home monitoring. Moreover, physicians had more experience than nurses (25% and 8% respectively).

**Figure 4:**
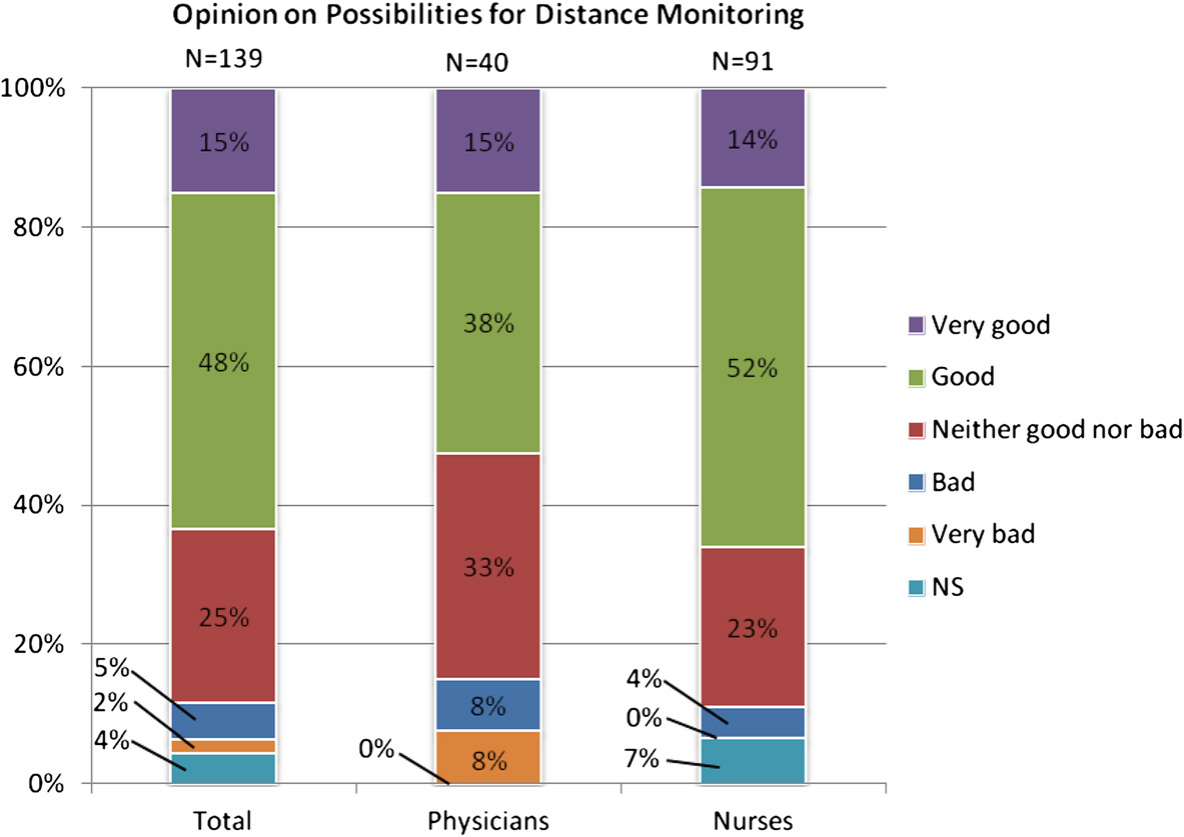
Possibilities for distance monitoring.

Figure 4 presents the answers to the question on possibilities for using distance monitoring in the care of patients. As the figure shows, a majority of the respondents believed that the possibilities are good or very good. One fourth of the respondents were neutral, leaving very few finding this to be a bad or very bad method.

**Figure 5:**
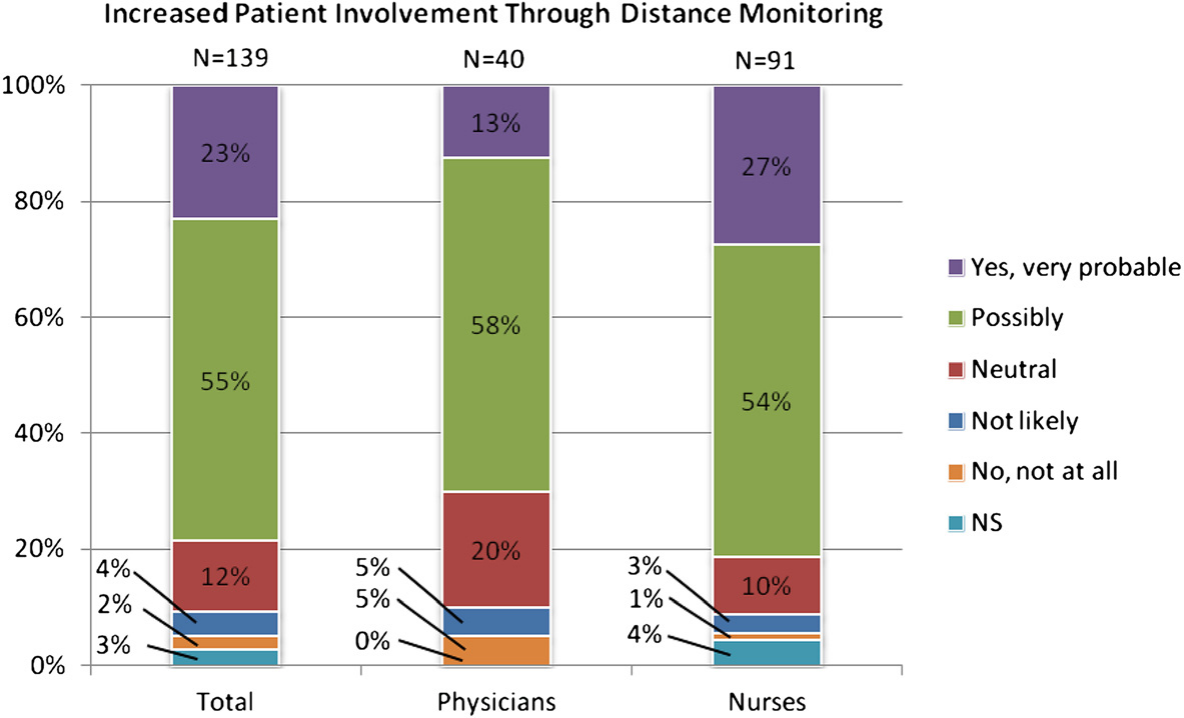
Attitudes among respondents regarding effect of distance monitoring on patient involvement.

**Figure 6:**
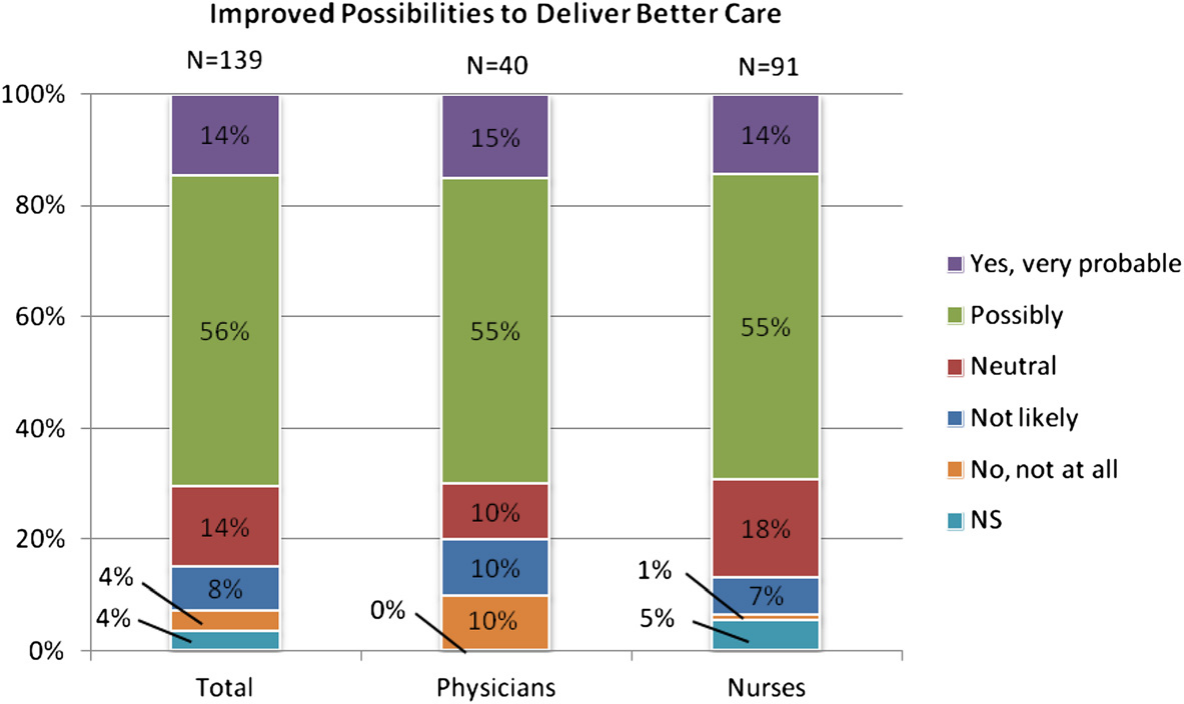
Attitudes among respondents regarding improved possibilities to deliver better care though distance monitoring.

According to more than three quarters of the respondents it is possible that patient involvement in the care process could change by means of distance monitoring, as shown in Figure 5. Furthermore, Figure 6 shows that 70% of the respondents believed that distance monitoring is likely or very likely to improve their own possibilities to deliver better care.

**Figure 7:**
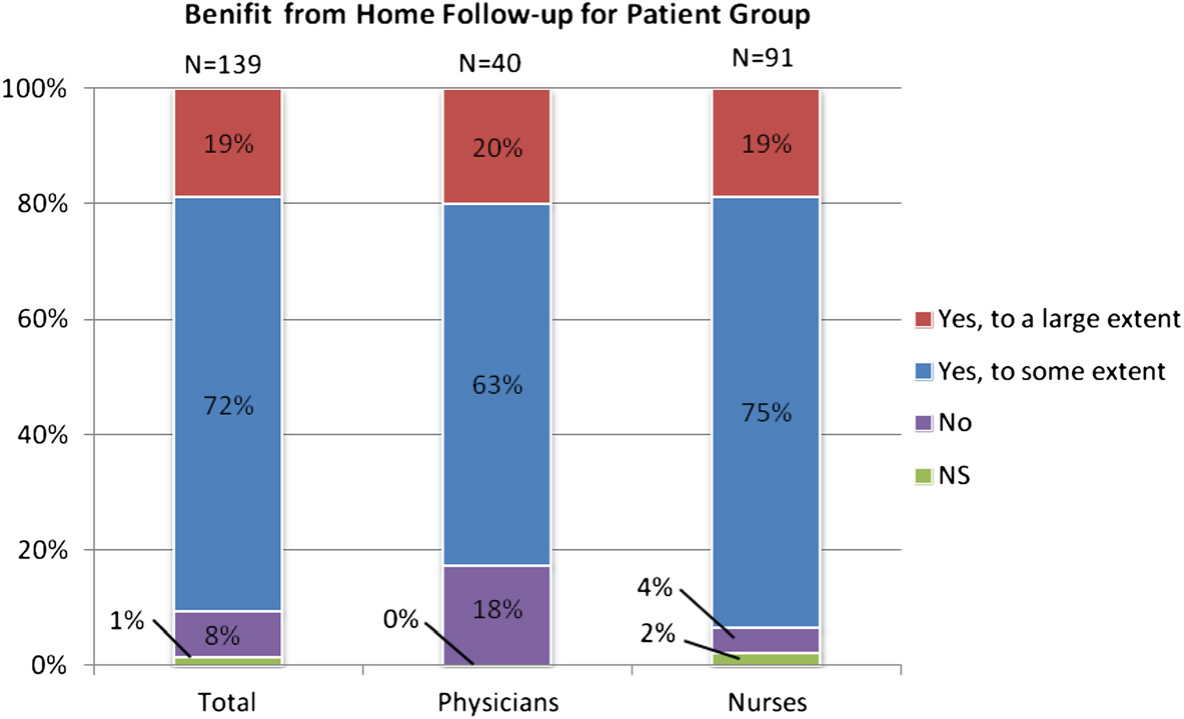
Attitudes on whether the respondents’ patient groups would benefit from follow-up at home.

As illustrated in Figure 7, most respondents thought that their patients (CHF) would benefit from home monitoring. However, most of them said to a certain level. Free text comments added to this question mostly concerned the patient’s high age and the likelihood that they are inexperienced in computers and technology. The need for a general impression of the patient was also mentioned. Other comments were problems related to not being able to measure certain parameters at home, the patient’s need of social contact, and that ICT systems for home monitoring are not yet clinically validated.

**Figure 8:**
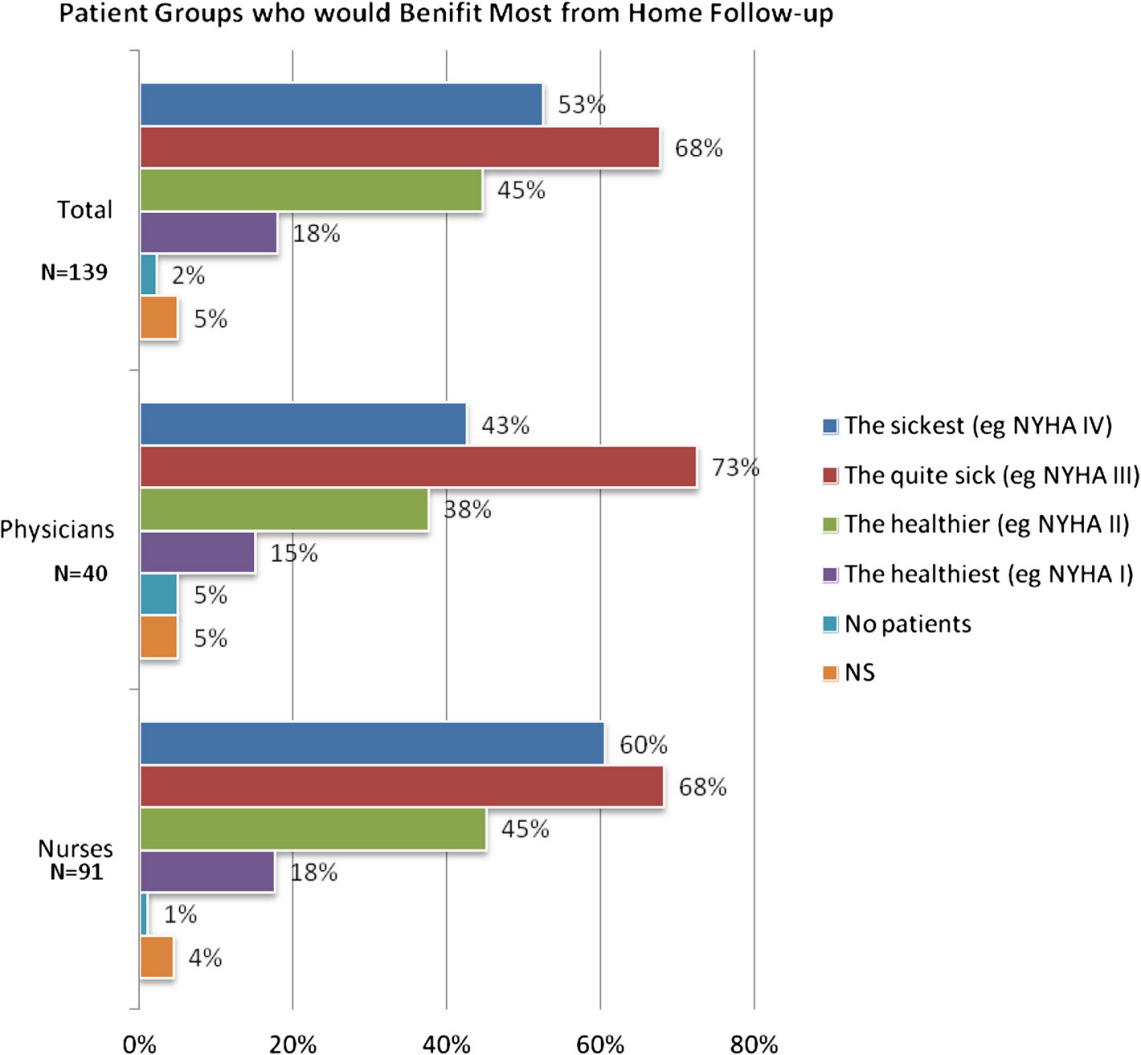
The patients who would benefit most from follow-up at home according to respondents.

Opinions on which patients with CHF that would benefit most from home monitoring can be seen in Figure 8. The quite sick patients would benefit most, closely followed by the very sick and the healthier patients. Only 2% did not think any patients would benefit from home monitoring.

Other suitable groups for home monitoring mentioned in comments were palliative patients, patients in initial medical adjustment, patients who would feel more secure, patients living far away or having difficulties reaching the hospital, healthy patients with cardiac disorders, and patients with ischemia. One comment mentioned that different methods of home follow-up could be applied to different patient groups depending on need.

**Figure 9:**
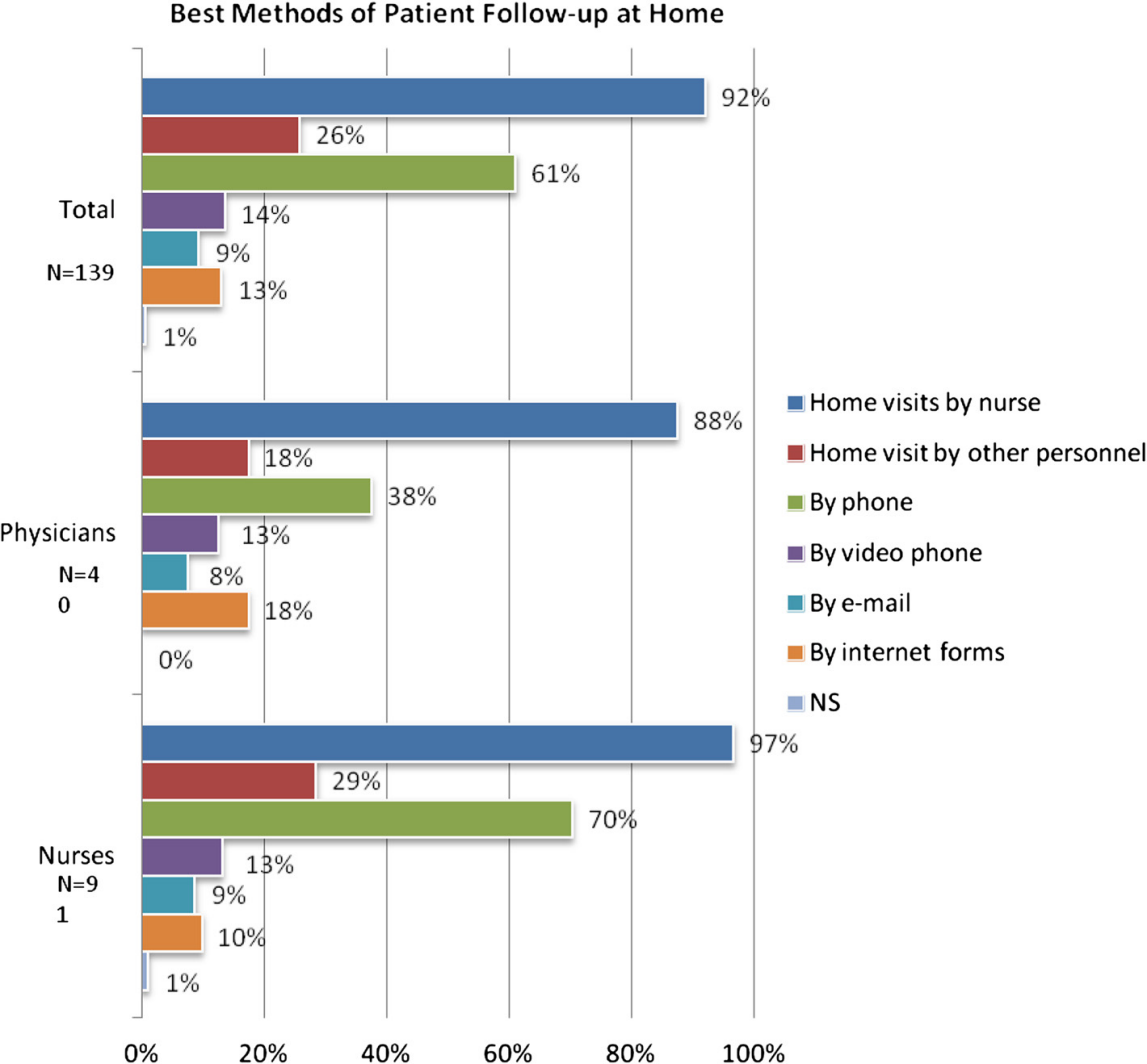
Best methods for following patients at home according to respondents.

Figure 9 shows that home visits by a nurse was considered to be the best way of monitoring patients, followed by phone contact. More technology based methods such as video telephony, Internet forms and e-mail were not as highly appreciated. More advanced monitoring of e.g. pacemakers were mentioned in the free text comments.

### Other

In the section “Other” the respondents were able to leave free text comments on their thoughts, ideas and experiences. Around 20% (31 respondents) gave comments. In conclusion, most comments were positive to ICT in healthcare, although cautious.

The comments varied between descriptions of experiences in the field, to advantages and drawbacks of home follow-up and technology. Common topics for good follow-up methods were home visits by physicians, nurses and/or home care personnel, telephone support, video telephony, e-mail and Internet tools. Among concerns were issues such as lack of personal contact with patient, ICT knowledge among elderly, current ICT systems at the clinic not working properly, subjective information not being reliable, and that some research in the area is inconclusive or negative to ICT tools for home follow-up. A few comments regarded that ICT tools could be a very good complement to personal contact, that distances and bad health could be a problem for patients when getting to the clinic and that younger patients might find this useful. Self-care and disease awareness was pointed out as being important.

### Differences between occupations

In general, physicians were more negative in their answers than nurses. An exception was future possibilities and time saving aspects where they gave roughly the same answers as the other groups. This is supported by results from a related study by Darr et. al. [26].

## Discussion

This paper describes a survey study performed at departments responsible for CHF care, e.g. cardiology and medicine departments, at Swedish hospitals. The results give an indication to the attitudes towards ICT in healthcare and the use of home monitoring within a selected group of healthcare professionals working with CHF patients.

### Dispatch and collection of data

Approximately one third of the questionnaires dispatched were collected in this study. If seen as a measure of response frequency, it can be considered to be quite low. Other studies have also shown the difficulty in obtaining high answering frequency in questionnaire surveys among healthcare personnel [27-29]. However, since we do not know how many of these questionnaires that were actually delivered to healthcare personnel and how many stayed in the hands of the heads of departments, i.e. were not distributed, we cannot consider this to be a measure of answering frequency. Moreover, when dispatching the questionnaires we assumed that 5 questionnaires per department was an adequate amount. The number of healthcare personnel is of course very dependent of the individual department; some might have very few personnel, some might have many. Some departments might not even have 5 staff members who could answer the questionnaire.

Another drawback of this type of dispatch is bias, as we ask each of the heads of the departments to distribute the questionnaires to selected personnel. This means that the head of the department may chose who answers the questionnaire depending on e.g. who in the staff is most interested in technology. In that case we could have a more positive response to ICT among the respondents than with the general staff population. Hence, our results should be considered to be indications on opinions within this actual group of healthcare professionals, and might not be applicable to the general population of healthcare professionals working in CHF care. Still, we believe that the results reflect and illustrate valuable attitude indicators within the targeted group.

It should also be noted that it was impossible to find relevant information on how many individuals could be considered to constitute the targeted group. In order to make future studies more accurate and reliable, a register of professionals working in specific areas of medicine would be beneficial. However, at date not even the national CHF specialist organisation is able to provide this information. Therefore alternative ways had to be explored to gather the information necessary for this study.

### Attitudes to ICT tools in healthcare

According to our results, the general opinions on ICT in healthcare today are positive, or even surprisingly positive, in the light of on-going discussions in various public media, where for instance negative comments on EPR etc. are fairly common. What is also interesting is that the opinions on the future of ICT are even more positive. Therefore, resistance from healthcare professionals to ICT in healthcare according to our findings should not be an issue for the implementation of ICT in healthcare. Although our results indicate positive attitudes, related studies have shown opposite results [7]. Whether this is due to differences in the respondent groups, targeted specialty, ICT experience or general attitude is difficult to say. It would therefore be of interest to further investigate the reasons for attitude related implementation issues in healthcare.

### Attitudes towards distance monitoring and home follow-up of patients

The majority of the respondents had no experience of distance monitoring. This means that the opinions of most respondents were based upon beliefs rather than experience. Lack of experience could have resulted in negative opinions on follow-up and monitoring at home, but our results show otherwise. Instead most respondents were positive to these methods.

The generally low rating of Internet forms as a method of home follow-up could be explained by the difficulty in picturing what such a system could look like, and how it should be utilized if experience and practical examples are lacking. Many studies and projects dealing with home monitoring of patients suffering from CHF have been based on monitoring of various vital sign parameters [11,30]. Therefore it is not unlikely that most respondents refer to this type of solution when answering the question. Jakob Nielsen discusses in his book “Usability Engineering” that although user opinions should always be considered it is often difficult for the users to know how to interact with systems they have no experience with [31]. This is well illustrated in the famous words of Henry Ford:

"“If I had asked people what they wanted, they would have said faster horses.”"

### Differences between occupations

The tendency that physicians are less positive towards home follow-up than nurses might be explained by the difference in working routines. For example, physicians and nurses might be interested in different types of information. Physicians might be more used to working with objective data (signs), while nurses prefer subjective data (symptoms). Working routines could also be the reason why physicians and nurses have different opinions on which patients are most suitable, as well as method of follow-up. It seems that physicians have less confidence in phone as a mean of following the patients. On the other hand, when it comes to using Internet forms, physicians are more positive than nurses.

A difference in computer usage at home can be seen between both physicians and nurses, as well as between men and women. Since the nurse group to a large majority consists of women, the question is whether it is the occupation (maybe length and type of education) or gender that is the reason for this difference. Numbers from the administrative agency Statistics Sweden show no major difference in computer and Internet usage among Swedish men and women in working age [32], indicating that the difference could be due to occupation rather than gender. Another possibility is that age is the reason as our results show that the responding nurses are “older” than the responding physicians, and an analysis of the material indicate that both computer usage at home as well as opinions on ICT in healthcare could be related to age.

### Introduction into clinical practice

Altogether our results indicate a general acceptance and positive attitude among healthcare personnel for ICT in healthcare and home follow-up of patients. Therefore the question on why the implementation of new eHealth tools, in routine daily care, often fail, still remains. One reason could be a reluctance to change working routines among the staff, something we have not investigated in this study. Maybe it is not the development they dislike; it is the change. When introducing the new tools one must also be prepared to change routines and maybe reallocate resources in order to optimize the workflow and the working methods in the new setting [33]. However, this might not be obvious to the staff beforehand, and it could be hard to realize how the new tool will be used based on the current routines. It may also be regarded as an “add-on” service to the patient, which cannot be handled within the existing routines. This stresses the importance of good change management, engagement and pre-marketing activities from management and healthcare decision makers.

Another reason for the difficulty in introducing new eHealth tools for management of chronic diseases could lie, not in resistance, but in the lack of interaction between hospital and primary care. Most research and development is taking place in hospital care, but many of the patients in question for these applications are being cared for in the primary care. The differences in organization and work method could be an issue as well as the “hand over/transition” procedures.

### Possible future studies

In order to gain further knowledge in the eHealth domain it could also be interesting to study a more general target group, as well as other specialties, such as neonatology, Parkinson’s, chronic obstructive pulmonary disease (COPD) and diabetes. And also to study the opinion of other groups such as patients and healthcare planners, executives, administrators and other healthcare decision makers at different levels in order to get a better view from the entire healthcare community.

## Conclusion

In this paper we have studied the opinions towards eHealth tools among healthcare professionals working with CHF patients at Swedish hospitals. The results indicate that the general opinions among the healthcare professionals in this study, on healthcare ICT are positive, as well as opinions on home follow-up and distance monitoring. People with chronic heart failure seem to be an appropriate group of patients for these methods. There are no major differences between physicians and nurses; however physicians tend to be more pessimistic than nurses. The reasons for reluctance to introduce new eHealth tools are still not evident after this study, but it seems that general resistance towards ICT or home monitoring in healthcare is not the major obstacle within the addressed group of healthcare personnel.

## Competing interests

BAS was at the time of the survey employed by Ortivus AB. There are no other known competing interests in this study.

## Authors’ contributions

AG designed the questionnaire together with KL, BAS and MS. She was also in charge of dispatching, collecting and analysing the questionnaires. Moreover, she was the main author of the manuscript. KL and BAS both supervised and were responsible for the project. They took part in all decisions on design and analysis, and were also the main mediators of funding. MS and HP took part in analysis and discussing regarding the results. All authors took part in writing, reading and approving the manuscript.

## Pre-publication history

The pre-publication history for this paper can be accessed here:

http://www.biomedcentral.com/1472-6947/12/138/prepub

## References

[B1] What is eHealth? http://ec.europa.eu/information_society/activities/health/whatis_ehealth/index_en.htm.

[B2] Eysenbach G What is e-health? J Med Internet Res 2001 3 e20 10.2196/jmir.3.2.e20 11720962 PMC1761894

[B3] Läkemedeldsverket (Swedish Medical Products Agency) Läkemedelsboken 2011-2012 2010 Sweden http://www.lakemedelsboken.se.

[B4] Jervall L Pehrsson T eHälsa i Landstingen, Landstingens IT-strateger/IT-chefer (SLIT-group) 2010 Sweden

[B5] Anderson JG Social, ethical and legal barriers to E-health Int J Med Inform 2007 76 480 483 10.1016/j.ijmedinf.2006.09.016 17064955

[B6] Sharma S Xu H Wickramasinghe N Ahmed N Electronic healthcare: issues and challenges Int J Electron Healthc 2006 2 50 65 10.1504/IJEH.2006.008693 18048234

[B7] Ward R Stevens C Brentnall P Briddon J The attitudes of health care staff to information technology: a comprehensive review of the research literature Health Info Libr J 2008 25 81 97 10.1111/j.1471-1842.2008.00777.x 18494643

[B8] Lapointe L Rivard S A multilevel model of resistance to information technology implementation MIS Q 2005 29 461 491

[B9] Gagnon M-P Desmartis M Labrecque M Car J Pagliari C Pluye P Frémont P Gagnon J Tremblay N Légaré F Systematic review of factors influencing the adoption of information and communication technologies by healthcare professionals J Med Syst 2010 36 1 37 20703721 10.1007/s10916-010-9473-4 PMC4011799

[B10] Kashem A Cross RC Santamore WP Bove AA Management of heart failure patients using telemedicine communication systems Curr Cardiol Rep 2006 8 171 179 10.1007/s11886-006-0030-1 17543243

[B11] Dickstein K Cohen-Solal A Filippatos G McMurray JJV Ponikowski P Poole-Wilson PA Strömberg A van Veldhuisen DJ Atar D Hoes AW ESC guidelines for the diagnosis and treatment of acute and chronic heart failure 2008 Eur J Heart Fail 2008 10 933 989 10.1016/j.ejheart.2008.08.005 18826876

[B12] Roger VL Go AS Lloyd-Jones DM Adams RJ Berry JD Brown TM Carnethon MR Dai S de Simone G Ford ES AHA statistical update: heart disease and stroke statistics - 2011 update Circulation 2011 123 e18 e209 10.1161/CIR.0b013e3182009701 21160056 PMC4418670

[B13] Dickstein K Cohen-Solal A Filippatos G McMurray JJV Ponikowski P Poole-Wilson PA Strömberg A Van Veldhuisen DJ Atar D Hoes AW ESC guidelines for the diagnosis and treatment of acute and chronic heart failure 2008 Eur Heart J 2008 29 2388 2442 18799522 10.1093/eurheartj/ehn309

[B14] Inglis SC Clark RA McAlister FA Ball J Lewinter C Cullington D Stewart S Cleland JGF Structured telephone support or telemonitoring programmes for patients with chronic heart failure Cochrane Database Syst Rev 2010 8 CD007228 Review. 20687083 10.1002/14651858.CD007228.pub2

[B15] Chaudhry SI Mattera JA Curtis JP P Spertus JA Herrin J Lin Z Phillips CO Hodshon BV Cooper LS Krumholz HM Telemonitoring in patients with heart failure N Engl J Med 2010 363 2301 2309 10.1056/NEJMoa1010029 21080835 PMC3237394

[B16] Dendale P De Keulenaer G Troisfontaines P Weytjens C Mullens W Elegeert I Ector B Houbrechts M Willekens K Hansen D Effect of a telemonitoring-facilitated collaboration between general practitioner and heart failure clinic on mortality and rehospitalization rates in severe heart failure: the TEMA-HF 1 (TElemonitoring in the MAnagement of Heart Failure) study Eur J Heart Fail 2012 14 3 333 340 10.1093/eurjhf/hfr144 22045925

[B17] Venter A Burns R Hefford M Ehrenberg N Results of a telehealth-enabled chronic care management service to support people with long-term conditions at home J Telemed Telecare 2012 18 3 172 175 22362838 10.1258/jtt.2012.SFT112

[B18] Seto E Leonard KJ Cafazzo JA Barnsley J Masino C Ross HJ Mobile phone-based telemonitoring for heart failure management: a randomized controlled trial J Med Internet Res 2012 14 1 e31 10.2196/jmir.1909 22356799 PMC3374537

[B19] Gund A On the Design and Evaluation of an eHealth System for Management of Patients in Out-of-Hospital Care. PhD Thesis 2011 Gothenburg, Sweden: Chalmers University of Technology

[B20] Seybert H Lööf A Internet usage in 2010 – Households and Individuals Data in focus, vol. 50/2010 2010 Eurostat: European Commission: European Union

[B21] Eurostat Individuals - Computer use 2010 European Commission. European Union

[B22] World Telecommunication/ICT Indicators Database Internet users 2010 Geneva, Switzerland: United Nations Statistics Division http://www.itu.int/ITU-D/ict/.

[B23] Care@Distancehttp://care-at-distance.com/.

[B24] Statistikdatabasen Sysselsatta 15–74 år (AKU) efter anknytningsgrad till arbetsmarknaden, näringsgren SNI2007 och kön. År 2009–2010 2010 Örebro, Sweden: Statistiska Centralbyrån (Statistics Sweden) http://www.ssd.scb.se/.

[B25] Löner och sysselsättning inom landstingskommunal sektor 2011 (Wages / salaries and employment in county councils 2011) 2012 Sweden: Statistiska Centralbyrån (Statistics Sweden)

[B26] Darr A Harrison M Shakked L Shalom N Physicians’ and nurses’ reactions to electronic medical records: managerial and occupational implications J Heal Organ Manag 2003 17 349 359 10.1108/14777260310505129 14628488

[B27] Hill CA Fahrney K Wheeless SC Carson CP Survey response inducements for registered nurses West J Nurs Res 2006 28 322 334 10.1177/0193945905284723 16585808

[B28] Barclay S Todd C Finlay I Grande G Wyatt P Not another questionnaire! Maximizing the response rate, predicting non-response and assessing non-response bias in postal questionnaire studies of GPs Fam Pract 2002 19 105 111 10.1093/fampra/19.1.105 11818359

[B29] Sobal J DeForge BR Ferentz KS Muncie HL Valente CM Levine DM Physician responses to multiple questionnaire mailings Eval Rev 1990 14 711 722 10.1177/0193841X9001400611

[B30] Clark RA Inglis SC McAlister FA Cleland JGF Stewart S Telemonitoring or structured telephone support programmes for patients with chronic heart failure: systematic review and meta-analysis Br Med J 2007 334 942 945 10.1136/bmj.39156.536968.55 17426062 PMC1865411

[B31] Jakob N Usability Engineering 1993 1 San Francisco, USA: Morgan Kaufmann

[B32] Privatpersoners användning av datorer och Internet 2010 (Use of computers and the Internet by private persons in 2010) 2011 Stockholm, Sweden: Statistiska Centralbyrån (Statistics Sweden)

[B33] Lamothe L Fortin J-P Labbe F Gagnon M-P Messikh D Impacts of telehomecare on patients, providers, and organizations Telemed J E-Health 2006 12 363 369 10.1089/tmj.2006.12.363 16796505

